# Herbal Medicines for Cardiovascular Diseases

**DOI:** 10.1155/2014/809741

**Published:** 2014-11-25

**Authors:** Xingjiang Xiong, Francesca Borrelli, Arthur de Sá Ferreira, Tabinda Ashfaq, Bo Feng

**Affiliations:** ^1^Department of Cardiology, Guang'anmen Hospital, China Academy of Chinese Medical Sciences, Beijing 100053, China; ^2^Department of Pharmacy, University of Naples Federico II, 80131 Naples, Italy; ^3^Laboratory of Human Motion Analysis, Augusto Motta University Center, 21041-021 Rio de Janeiro, RJ, Brazil; ^4^Department of Family Medicine, Aga Khan University, Karachi 74800, Pakistan

The global burden of disease has driven a broad shift from communicable, maternal, neonatal, and nutritional causes to noncommunicable diseases. Cardiovascular diseases (CVDs) remain the most prevalent cause of human morbidity and mortality all over the world [1]. According to the survey by Global Burden of Disease Study, 29.6% of all deaths worldwide were caused by CVDs in 2010 [2]. It is estimated that the number of people that die from CVDs, mainly from heart disease and stroke, will increase to more than 24 million by 2030 [3]. Despite progress in molecular medicine and biology and translational scientific efforts on improvement of diagnostic and therapeutic strategies over the past 20 years, CVDs continue to be a major global health problem.

The use of herbal medicines, one of the main therapeutic approaches of complementary and alternative medicine (CAM), can be tracked back thousands of years ago in the East [4]. Currently, there is a recent resurgence of the use of herbal medicines in popularity among patients in the West and they were consumed by more than 15 million people in the US [5]. With increasing enhancement of people's awareness of self-care and concerning on the inevitable adverse effects of conventional medicine, herbal medicines are favored by people with CVDs all over the world for their unique advantages in preventing and curing diseases, rehabilitation, and health care [6]. There is growing evidence showing that many herbal medicines and their active ingredients contribute to the standard therapy for CVDs, for example, aspirin, digitalis, and reserpine [7].

Despite enormous interests in the medicinal uses by consumers, there is still a great deal of confusion and misunderstanding about their identification, effectiveness, pharmacology, toxicology, and herb-drug interaction to science world [8]. Therefore, the role of herbal medicines in CVDs still needs more scientific and clinical data proving their efficacy and safety. The special issue aims to summarize the current progress of promising herbal medicines and their extractions for various CVDs.

Altogether, we gathered 31 papers for publication, out of which 14 papers were accepted. The original research articles and reviews in this issue cover a wide range of topics, including coronary heart disease, hypertension, heart failure, dyslipidemia, and arrhythmia. Five papers addressed the clinical application and the mechanism of herbal medicines in the treatment of coronary heart disease. “*A multicentre randomized clinical trial on efficacy and safety of huxin formula in patients undergoing percutaneous coronary intervention*” provided evidence on* huxin* formula, an experienced Chinese medicine formula, for the treatment of patients undergoing percutaneous coronary intervention. “*Traditional formula, modern application: Chinese medicine formula sini tang improves early ventricular remodeling and cardiac function after myocardial infarction in rats*” evaluated the improvement of early ventricular remodeling and cardiac function in myocardial infarction in rats by* sini tang*, which is a traditional Chinese classical herbal formula first described by Zhongjing Zhang (150–219 A.D.). “*The comparative study on expression of SIRT1 signal transduction by xuefuzhuyu capsule*” tested the protective effect of* xuefuzhuyu* formula, another classical herbal formula in traditional Chinese medicine (TCM), on ischemic myocardial cells induced by ischemia through SIRT1-mediated signal transduction pathway. “*Protective effects of shen-yuan-dan, a traditional Chinese medicine, against myocardial ischemia/reperfusion injury in vivo and in vitro*” investigated the effectiveness and mechanisms of* shen-yuan-dan*'s pharmacological postconditioning on myocardial ischemia/reperfusion injury by targeting the phosphatidylinositol 3-kinase/Akt (PI3K/Akt) pathway. “*Ligusticum wallichii extract inhibited the expression of IL-1*β* after AMI in rats*” addressed the effects of* Ligusticum wallichii *(chuanxiong) extract on IL-1*β*expression in myocardium and central nervous system after acute myocardial infarction.

Hypertension is an important public-health challenge worldwide and a major risk factor for stroke, myocardial infarction, vascular disease, and chronic kidney disease. Prevention, detection, treatment, and control of this condition should receive high priority. How about the role of TCM for managing hypertension? One review article “*Traditional Chinese medicine syndromes for essential hypertension: a literature analysis of 13,272 patients*” analyzed the diagnosis rules and common TCM syndromes of hypertension and recommended the corresponding Chinese herbal medicines and formulas. “*Chinese herbal medicine bushen qinggan formula for blood pressure variability and endothelial injury in hypertensive patients: a randomized controlled pilot clinical trial*” examined the therapeutic effects of* bushen qinggan *formula as adjunctive therapy for antihypertensive drugs on mean blood pressure, blood pressure variability, and endothelial function for hypertension.

One paper “*Yiqi huoxue recipe improves heart function through inhibiting apoptosis related to endoplasmic reticulum stress in myocardial infarction model of rats*” explored the mechanism of cardioprotective effects of* yiqi huoxue* formula in rats with myocardial infarction-induced heart failure by inhibiting endoplasmic reticulum stress response pathway.

Two papers discussed the cardiovascular protective effects of Hawthorn (*Crataegus oxyacantha*). “*Effect of Crataegus usage in cardiovascular disease prevention: an evidence-based approach*” reviewed the cardiovascular pharmacological properties of* Crataegus in vivo* and* in vitro*. “*Evaluation of a Crataegus-based multiherb formula for dyslipidemia: a randomized, double-blind, placebo-controlled clinical trial*” examined the effects of a multiherb formula containing* Crataegus *pinnatifida on plasma lipid and glucose levels in Chinese patients with dyslipidemia.

Finally, “*Yiqihuoxuejiedu formula inhibits vascular remodeling by reducing proliferation and secretion of adventitial fibroblast after balloon injury*” analyzed effects and mechanisms of the* yiqihuoxuejiedu* formula on inhibiting vascular remodeling, especially adventitial remodeling. “*Ganoderma lucidum polysaccharides reduce lipopolysaccharide-induced interleukin-1*β* expression in cultured smooth muscle cells and in thoracic aortas in mice*” examined the effects of an extract of* Ganoderma lucidum* (Reishi) polysaccharides on interleukin-1*β* expression by human aortic smooth muscle cells (HASMCs) and the underlying mechanism. A review article “*Aspirin resistance and promoting blood circulation and removing blood stasis: current situation and prospectives*” provided insight into the relationship between aspirin resistance and blood stasis syndrome and explored the therapeutic role of Chinese herbal medicines with promoting blood circulation and removing blood stasis for this condition.

Recently, a great progress has been made focusing on the effectiveness and safety of herbal medicines in patients with CVDs. Some RCTs and systematic reviews provided strong evidence for clinical usage. The special issue presented the updated knowledge of partial herbal medicines for CVDs, which unraveled a complex posttranscriptional gene-regulating machinery and paved the evidence-based way.

*Xingjiang Xiong* *Xingjiang Xiong* *Francesca Borrelli* *Francesca Borrelli* *Arthur de Sá Ferreira* *Arthur de Sá Ferreira* *Tabinda Ashfaq* *Tabinda Ashfaq* *Bo Feng* *Bo Feng*

## References

[1] Nichols M., Townsend N., Scarborough P., Rayner M. (2014). Cardiovascular disease in Europe 2014: epidemiological update. *European Heart Journal*.

[2] Lozano R., Naghavi M., Foreman K. (1990). Global and regional mortality from 235 causes of death for 20 age groups in 1990 and 2010: a systematic analysis for the Global Burden of Disease Study 2010. *The Lancet*.

[3] Fuster V. (2014). Global burden of cardiovascular disease: time to implement feasible strategies and to monitor results. *Journal of the American College of Cardiology*.

[4] Liu X., Wu W. Y., Jiang B. H., Yang M., Guo D. A. (2013). Pharmacological tools for the development of traditional Chinese medicine. *Trends in Pharmacological Sciences*.

[5] Eisenberg D. M., Davis R. B., Ettner S. L., Appel S., Wilkey S., Van Rompay M., Kessler R. C. (1998). Trends in alternative medicine use in the United States, 1990–1997: results of a follow-up national survey. *Journal of the American Medical Association*.

[6] Tachjian A., Maria V., Jahangir A. (2010). Use of herbal products and potential interactions in patients with cardiovascular diseases. *Journal of the American College of Cardiology*.

[7] Xiong X., Yang X., Liu Y., Zhang Y., Wang P., Wang J. (2013). Chinese herbal formulas for treating hypertension in traditional Chinese medicine: perspective of modern science. *Hypertension Research*.

[8] Izzo A. A., di Carlo G., Borrelli F., Ernst E. (2005). Cardiovascular pharmacotherapy and herbal medicines: the risk of drug interaction. *International Journal of Cardiology*.

